# Suitable Habitat Distribution and Niche Overlap of the Sable (*Martes zibellina*) and Yellow-Throated Marten (*Martes flavigula*) in Taipinggou National Nature Reserve, Heilongjiang Province, China

**DOI:** 10.3390/biology13080594

**Published:** 2024-08-07

**Authors:** Yang Hong, Xinxin Liu, Ning Zhang, Ziwen Wang, Changzhi Zhang, Minghai Zhang

**Affiliations:** 1 College of Wildlife and Protected Area, Northeast Forestry University, Harbin 150040, China; hy1624@126.com (Y.H.); liuxinxin2017@126.com (X.L.); 18846797329@163.com (N.Z.); 15008225571@163.com (Z.W.); 2 Jiangxi Environmental Engineering Vocational College, Ganzhou 341000, China

**Keywords:** biodiversity, interspecies competition, niche overlap, sable (*Martes zibellina*), yellow-throated marten (*Martes flavigula*)

## Abstract

**Simple Summary:**

We used the line transect method and infrared camera traps to research the sable and yellow-throated marten in Taipinggou National Nature Reserve, Heilongjiang, China, to understand the distribution and overlap of their habitats and niches. In our study, the results showed that the habitat suitable for sables was larger than that for yellow-throated martens and that there is a partial overlap in terms of the suitable habitat for both species. There was a high degree of niche overlap between the sable and yellow-throated marten, primarily influenced by four environmental variables, i.e., “Distance from Settlements”, “Distance from Roads”, “Slope”, and “Distance from Coniferous and Broadleaved Mixed Forest”. This study can offer valuable insights for the development of policies aimed at conserving and managing sables and yellow-throated martens.

**Abstract:**

The global focus on fostering harmonious interactions and promoting rational coexistence among wildlife species to uphold or reinstate biodiversity remains a prominent area of interest. We conducted a study on the sable and yellow-throated marten in Taipinggou National Nature Reserve, Heilongjiang, China, using the line transect method and infrared camera traps from 2022 to 2023. We then analyzed the overlap of their suitable habitats and niches with the aim of gaining insight into the interspecific competition between these two species. We found that the suitable habitat areas for the sable and yellow-throated marten were 55.20 km^2^ and 23.28 km^2^, accounting for 24.86% and 10.48% of the total area of this study, respectively. The overlap between the suitable habitats for the sable and yellow-throated marten was 15.73 km^2^, accounting for 28.5% and 67.6% of their suitable habitat, supporting our Hypothesis 1. The first principal component (Dim1) of the niche explained 35.4% of the overall variability, which is mainly related to the environmental variables “Distance from Settlements” and “Distance from Roads”. Overall, 25.5% of the total variability was explained by the second principal component (Dim2), associated with “Slope” and “Distance from Coniferous and Broadleaved Mixed Forest”. The niches occupied by the sable and yellow-throated marten were both off-center of the environmental background space, with the niches of the sable being larger than those of the yellow-throated marten. Schoener’s D index was 0.56, indicating a high degree of niche overlap between the sable and yellow-throated marten, supporting our Hypothesis 2. Our study is helpful in terms of formulating conservation and management policies for the sable and yellow-throated marten.

## 1. Introduction

Currently, there is a global trend of declining biodiversity [1]. The decline or potential extinction of certain species can be attributed to hunting by humans and the destruction of habitats, while other species may struggle to coexist with different species in the context of the rapidly changing climate, leading to population suppression or natural elimination [2,3,4]. The possibility of interspecies coexistence within the same distribution area depends on the healthiness of interactions between them. Imbalances in these interactions can disrupt ecological equilibrium, as exemplified by Asian carp in the United States [5,6], European rabbits (*Oryctolagus cuniculus*) in Australia [7,8], etc. In northeastern China, the cold climate contributes to a lower level of biodiversity compared to tropical and subtropical regions, thereby increasing the risk of biodiversity loss. Urgent measures are required to preserve or restore biodiversity in this area.

Habitat plays a crucial role in the survival and reproduction of wildlife. Accurately predicting the distribution of suitable habitats for species is essential in formulating effective protection and management policies. Selecting an efficient habitat prediction model is vital in accurately reflecting the real situation in a study area. Commonly used models for suitable habitat prediction include CLIMate Expertise, the Random Forest model, and the MaxEnt model [9,10]. Among these models, the MaxEnt model stands out due to its ability to handle nonlinear relationships between environmental factors with minimal data requirements—only species distribution and environmental factor data are needed. Therefore, it has a strong predictive capability. It has been widely applied in predicting suitable habitats for various wildlife species, such as the Amur tiger (*Panthera tigris altaica*) [11] and giant panda (*Ailuropoda melanoleuca*) [12].

Interactions among species primarily encompass competition, predation, and parasitism [13]. Interspecific competition represents a significant ecological interaction between species. According to Gause’s Hypothesis, the greater the similarity between two species in their resource utilization, the more intense their competition will be [14]. The sable (*Martes zibellina*) and the yellow-throated marten (*Martes flavigula*) coexist in the same geographical region, both belonging to the Mustelidae family and Martes genus within the Carnivora order [15]. The sable is a solitary animal with a body length of about 40 cm and a weight of approximately 1 kg, while the yellow-throated marten is a gregarious animal with a body length of approximately 56–65 cm and a weight of about 2–3 kg. The larger body size and the gregarious lifestyle give the yellow-throated marten an advantage over the purple marten. The coexistence of the sable and yellow-throated marten is the result of the exclusion of the smaller species by the larger species. If no research is carried out between them, then the end result is likely to be the complete exclusion of the sable from the ecosystem by the yellow-throated marten [16]. Moreover, both sables and yellow-throated martens serve as intermediate predators within the study area and are the primary predators of local small and medium-sized rodents. They actively contribute to regulating the populations of apex predators in the region and mitigating rodent infestations. Investigating the interspecific competition between sables and yellow-throated martens holds significant implications in comprehending species coexistence mechanisms, maintaining the health and equilibrium of the local ecological system, and enhancing biodiversity.

Based on the principle of competitive exclusion, two species with overlapping niches in the same area cannot coexist indefinitely. In such circumstances, these species will inevitably differentiate their ecological niches due to intense competition [17]. In 1958, Hutchinson provided a quantitative depiction of the niche by introducing the concept of niche space, which illustrates the range within which a species can survive or reproduce based on multiple environmental variables (i.e., niche dimensions). Hutchinson denoted a niche space with more than three dimensions as a hypervolume niche [18]. While they are mathematically representable, visually displaying hypervolume niches using graphs poses challenges due to their high dimensionality. To tackle this issue, we employed principal component analysis (PCA) to reduce the dimensionality of environmental variables and depict the species’ niche in a two-dimensional space. Subsequently, we quantified and visualized the species’ niche based on this analysis.

Habitats are the foundation of a species’ survival. Resource availability within a specific area is constrained, and as the degree of habitat overlap among species increases, the competition for resources becomes more intense [19]. Analyzing the distribution of co-occurring species and assessing the extent of habitat overlap can directly reflect the level of species competition. The niche reflects the functional relationship of each individual within a population or community, and niche overlap is closely associated with interspecific competition. Based on the principle of competitive exclusion and considering the survival characteristics of sables and yellow-throated martens, we proposed the following hypotheses: (1) there is an overlap between the suitable habitats for martens and yellow-throated martens, and (2) the degree of niche overlap between sables and yellow-throated martens is relatively substantial. Our study examines the distribution of suitable habitats and niche overlap between sables and yellow-throated martens in Taipinggou National Nature Reserve. The objective is to offer theoretical underpinning for the formulation of conservation and management strategies for sables and yellow-throated martens.

## 2. Materials and Methods

### 2.1. Study Area

**Figure 1:**
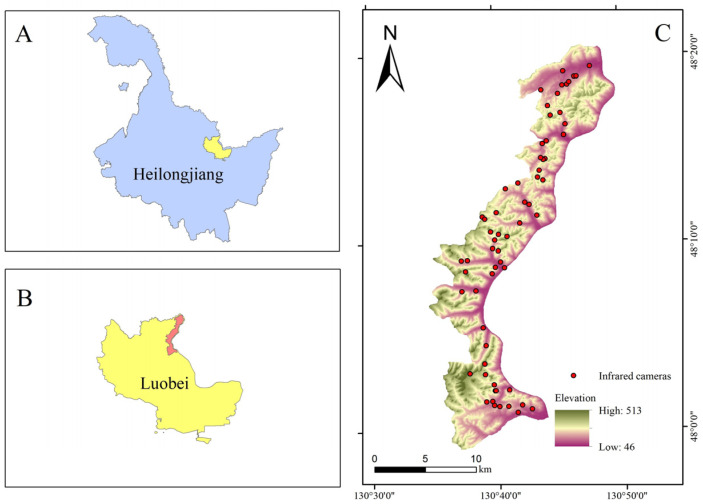
Location of Taipinggou Nature Reserve (**C**), Luobei country (**B**), Heilongjiang Province (**A**), China, and the deployment site of infrared camera traps.

The Taipinggou Nature Reserve (48°02′48″~48°20′19″ N, 130°31′12″~130°50′11″ E) is situated in Heilongjiang Province, northeast China (Figure 1), encompassing a total area of 221.99 km^2^. The elevation ranges from 46 to 513 m, and the region experiences a continental monsoon climate with an average annual temperature of 1 °C [20]. This reserve is designated as a China–Russia biodiversity international protection area and serves as one of the crucial cross-border ecological corridors for Amur tigers between China and Russia. The reserve boasts abundant wildlife and plant resources, characterized by a predominant coniferous forest consisting of *Picea asperata*, *Abies fabri*, *Pinus koraiensis*, etc. Notable wildlife in this area includes *Panthera tigris altaica*, *Ursus arctos*, and *Martes zibellina,* among other species [21]. Owing to rampant poaching and extensive forest degradation, the populations of sables and yellow-throated martens in Taipinggou Nature Reserve have been steadily dwindling. Urgent measures are imperative, such as undertaking comprehensive research on these species and formulating corresponding conservation policies to avert further decline.

### 2.2. The Occurrence Sites of the Sable and Yellow-Throated Marten

Line transects and infrared cameras were used to monitor the occurrence sites of the sable and yellow-throated marten. We randomly set up line transects in the study area and searched for signs of the sable and yellow-throated marten, including individuals, tracks, and feces, along the transects. When we found suspected signs of them, we recorded the coordinates and elevation of the site and also took photographs. After the survey, we identified the signs photographed using the “Field Guide to Mammalian Fauna of China”, “Guide to Wildlife Tracks of the Far East”, and other relevant works from the literature [22,23,24,25]. A total of 332 sable sites and 44 yellow-throated marten sites were identified through line transect surveys. 

Considering the biological and ecological characteristics of the sable and yellow-throated marten, infrared cameras were installed at passageways frequented by wildlife or in places with intensive traces of their activities, such as water sources, feeding points, animal marking places, courtship grounds, fallen logs, or forest roads. Sixty infrared cameras were deployed in our study. Each camera was spaced at least 1 km away from any other, with a wide and unobstructed view. The lens was kept horizontal or tilted slightly downwards. The cameras are programmed to operate continuously, capturing two images with a 2 s interval and one 10 s video once triggered [26,27]. We replaced the batteries and SD cards bimonthly [28]. Species identification was carried out for each photograph obtained. From December 2022 to June 2023, all cameras were operational for 3923 days in total. Fourteen cameras recorded images of the sable, while the yellow-throated marten was captured by three cameras. 

A total of 346 occurrence sites for sables and 47 for yellow-throated martens were identified. When the amount of wildlife does not reach the environmental carrying capacity, the quantity of traces can reflect the population abundance. However, if the environmental carrying capacity is exceeded, then the distribution of the species will be affected, which will, in turn, cause bias in the results of the model prediction. The Taipinggou Nature Reserve, which is under strict protection, exhibits a high environmental carrying capacity. Currently, the populations of sables and yellow-throated martens have not exceeded the environmental carrying capacity and will not introduce bias into the model’s predictions. To mitigate the impact of spatial autocorrelation on habitat distribution modeling, we utilized ENMTools to eliminate duplicate occurrence sites of the same species within each grid (30 m × 30 m) [29,30]. Ultimately, the number of sites used for sable and yellow-throated marten habitat distribution modeling was 270 and 35, respectively.

### 2.3. Environment Variables

Based on the vegetation types, climates, and other relevant factors in Taipinggou Nature Reserve, combined with the biological and ecological characteristics of the sable and yellow-throated marten, we selected 31 environmental variables for habitat distribution modeling and niche overlap analysis (Table 1) [31,32,33]. To eliminate the impact of strong covariance between environmental variables on habitat distribution modeling, Pearson correlation analyses were conducted on 31 environmental variables using the band-set statistics tool in ArcGIS 10.8.1, and correlation coefficients ∣r∣ < 0.8 were retained [34,35,36]. Following screening, nineteen environmental variables satisfied the criteria, including Elevation, Slope, Slope Aspect, Distance from Road (DR), Distance from Settlements (DS), Distance from Main Stream (DMS), Distance from Tributary (DT), Distance from Meadow (DM), Distance from Broadleaved Forest (DBF), Distance from Coniferous and Broadleaved Mixed Forest (DCBMF), NDVI, Annual Mean Temperature (Bio1), Mean Diurnal Range (Bio2), Isothermality (Bio3), Mean Temperature of Driest Quarter (Bio9), Annual Precipitation (Bio12), Precipitation of Wettest Month (Bio13), Precipitation of Driest Month (Bio14), and Precipitation Seasonality (Bio15). 

### 2.4. Habitat Distribution Modeling

The MaxEnt model is currently extensively employed in the prediction of habitat distribution for species such as roe deer (*Capreolus pygargus*) and Amur tigers [37,38]. Firstly, the processed species occurrence sites and environmental variables are imported into MaxEnt3.4.4. The options “Creating response curves”, “Make pictures of prediction”, and “Do jackknife to measure variable importance” are selected on the interface to plot response curves and assess the importance of each environmental variable. There are four output modes for the model (i.e., Cloglog, Logistic, Cumulative, and Raw), and we chose Logistic as the output format. The chosen output type of the file was in the ACSII format [32,33]. We allocated 80% of the species occurrence points to the training set for the model and reserved 20% for the test set. The model was run 10 times consecutively, and the average value calculated from these runs was used as the final result. The sub-sample was selected for the repeat run category. The model accuracy was assessed by studying the area under the characteristic curve (AUC), where a threshold of 0.80 or above indicates a favorable model performance [39,40]. The true skill statistic (TTS) was also used to assess the effectiveness of the model. The evaluation criteria were as follows: poor (0.2–0.5), effective (0.5–0.8), and excellent (>0.8) [41,42]. We employed the Jackknife test to analyze the importance of each environmental variable to the optimal model. The maximum training sensitivity plus the specificity logistic threshold was used to classify habitats as either suitable or unsuitable [43].

### 2.5. Analysis of Niche Overlap

The steps of niche overlap analysis include (1) principal component analysis (PCA) of environmental variables, (2) constructing the niche space, and (3) calculating the niche overlap index. PCA is a dimensionality reduction method that facilitates a comparison of the extent of niche overlap and disparity between two species within the environmental background space [44,45,46]. We randomly generated 5397 pseudo-absent points of sables and yellow-throated martens within the study area to serve as environmental background points for subsequent analysis. Based on the importance of each environmental variable output by the MaxEnt optimal model, we calculated the square root of the sum of the squares (SRSSs) of the importance of each environmental variable for the sable and the yellow-throated marten and then sorted the environmental variables in descending order based on this value. Subsequently, we eliminated the environmental variables one by one in descending order and performed PCA on the remaining ones after each elimination. At this stage, we selected two principal components with a cumulative variance contribution rate greater than 85% or a third principal component with an eigenvalue of less than 1. Finally, the obtained two principal components were used to construct a two-dimensional environmental background space [47]. The 2D environmental background space was gridded into a grid space of r × r resolution, and kernel functions were used to smooth the density of species occurrence points within each grid to visualize the 2D environmental background space, as well as the niches occupied by the species [48,49]. Schoener’s D index was used to evaluate the niche overlap among species, yielding values within the range of 0–1, where a higher value indicates a greater degree of overlap [50]. Data analysis was conducted in R.

## 3. Results

### 3.1. Habitat Distribution and Overlap

The mean AUCs of 10 replicate iterations of MaxEnt modeling for the sable and yellow-throated marten were 0.858 and 0.802, and the maximum TTS values were 0.796 and 0.784, respectively. The top three contributing variables to the sable modeling were Slope, Distance from Road, and Distance from Main Stream, which accounted for 60.4% of the total contributions. Correspondingly, Distance from Road, Distance from Coniferous and Broadleaved Mixed Forest, and Mean Diurnal Range were the three primary contributing factors for the yellow-throated marten; however, they only collectively explained 43.7% of the total contribution (Table S1).

**Figure 2:**
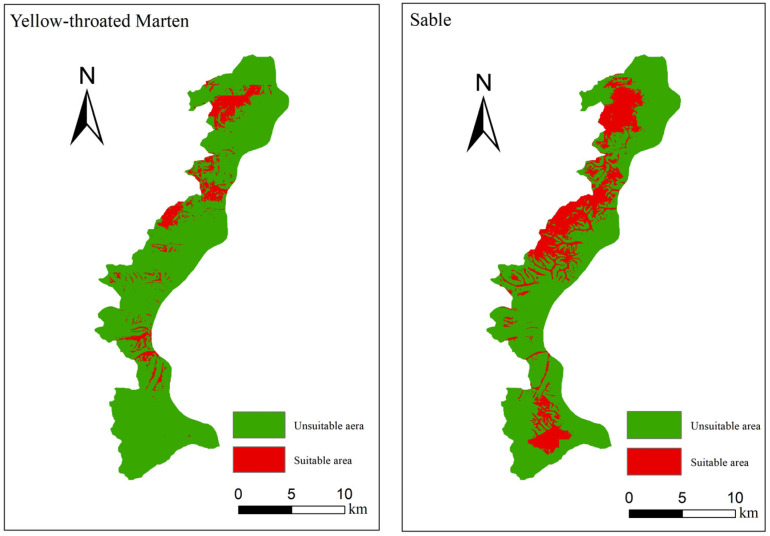
Distribution of suitable and unsuitable habitats for the sable and yellow-throated marten.

The suitable habitat area for the sable constitutes 55.20 km^2^, while that for the yellow-throated marten is 23.28 km^2^, and these areas represent 24.86% and 10.48% of Taipinggou Nature Reserve. The overlapping suitable habitat area for the sable and yellow-throated marten amounts to 15.73 km^2^, accounting for 28.5% and 67.6% of their respective suitable habitat areas (Figure 2), supporting Hypothesis 1.

### 3.2. Niche Overlap

**Figure 3:**
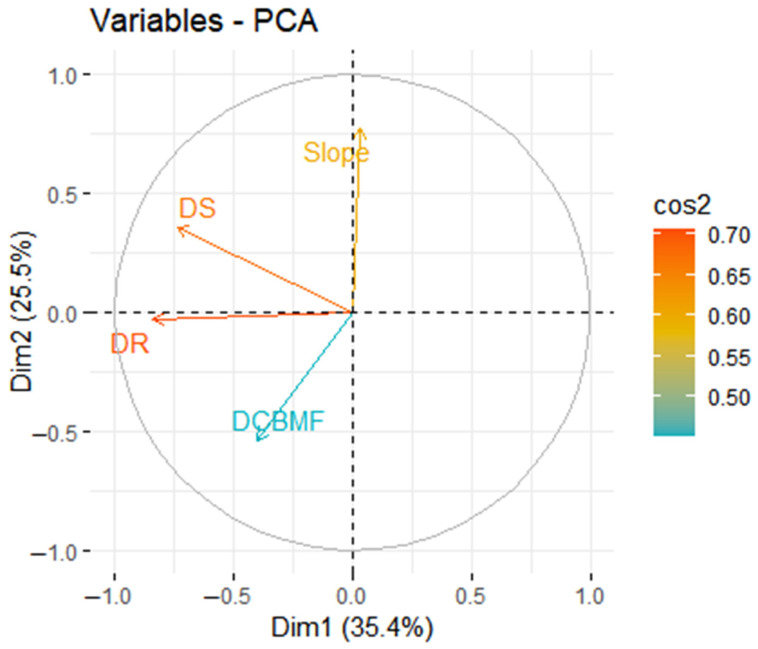
Correlation circle for the 16th round of PCA analyses of environmental variables. The cos2 indicates the explanatory power of the first two principal components (Dim1 and Dim2) for each environmental variable.

The SRSS of the importance of Slope was the largest (33.4), and that of Distance from Broadleaved Forest was the smallest (0.3) in all variables (Table 2). Commencing from DBF, the environmental variables with the lowest rankings were sequentially eliminated, and PCA was conducted on the remaining ones. Following the 16th round of PCA, the cumulative variance contribution rate of the first and second principal components reached 60.87%, while the eigenvalue of the third principal component was only 0.98 (<1). The first and second principal components exhibited sufficient explanatory power for overall environmental variables. Therefore, the four environmental variables of Slope, DR, DCBMF, and DS were used in the subsequent quantitative analysis of niches (Table 2). The first principal component (Dim1) accounted for 35.4% of the overall variability, mainly related to the DS and DR, and 25.5% of the total variability was explained by the second principal component (Dim2), associated with the Slope and DCBMF (Figure 3). 

**Figure 4:**
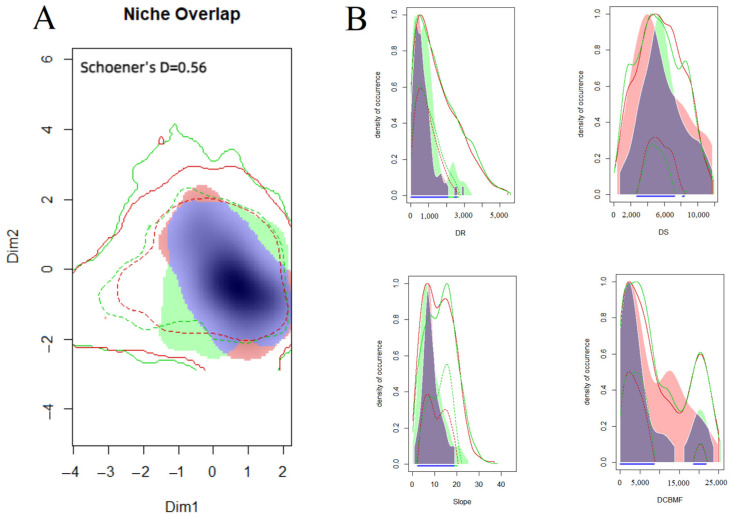
(**A**) The niche overlap between sables and yellow-throated martens in a 2D environment. Solid green and red lines represent 100% of the available environmental background space in the study area for the sable and yellow-throated marten, and dotted green and red lines represent 50% of the available environmental background space for the sable and yellow-throated marten. The green side shows the niche occupied by the sable, the red side shows the niche occupied by the yellow-throated marten, and the blue side shows the overlap between the sable and the yellow-throated marten. (**B**) The overlap between the sable and yellow-throated marten for each variable. The meanings of the lines and graphic colors are the same as in (**A**).

The niches occupied by the sable and yellow-throated marten were both situated away from the center of the environmental background space, with the sable occupying larger niches than the yellow-throated marten. Schoener’s D index was 0.56, which indicated a substantial degree of niche overlap between sables and yellow-throated martens (Figure 4A), supporting Hypothesis 2. The distribution density of the sable for DR exhibited primary and secondary peaks around 200 m and 800 m, and the yellow-throated marten had one primary peak around 400 m. The peaks of the distribution density of DS were around 5800 m and 4000 m, and those of Slope were around 6° and 8° for the sable and the yellow-throated marten, respectively. Concerning DCBMF, both the sable and yellow-throated marten had two distribution density peaks. The primary peaks both occurred around 2500 m; additionally, there was a secondary peak at 20,000 m for the sable and at 12,500 m for the yellow-throated marten. Although the main peaks of the two species were similar, yellow-throated martens demonstrated a greater tolerance for DMCBF compared to sables (Figure 4B).

## 4. Discussion

The survival of species is shaped by numerous external environmental factors, and elucidating the interplay between species and their environment constitutes a primary focus of ecological research [51,52]. We found that the first three variables affecting the habitat distribution of the sable accounted for 60.4 of the total contribution, while, for the yellow-throated marten, they accounted for only 43.7. This implies that the habitat distribution of sables is more susceptible to a few key factors. In contrast, the habitat distribution of the yellow-throated marten is influenced by a much larger number of environmental variables working together. Consequently, when there are significant changes in key factors, sables are more likely to experience adverse effects, whereas yellow-throated martens may demonstrate relatively lower susceptibility under similar circumstances. It is evident that the sable exhibits lower resilience to environmental changes compared to the yellow-throated marten, resulting in a greater impact on survival and a more restricted distribution range.

The sable and yellow-throated marten are not completely spatially differentiated (i.e., there is a partial spatial overlap), which may be influenced by their ecological traits and dietary preferences. Relevant studies have shown that the sable commonly inhabits mixed coniferous and broadleaved forests at an altitude of 800–1600 m and prefers to be active in the middle and at the bottom of slopes [53,54]. The yellow-throated marten was found at altitudes ranging from 428 to 3027 m, favoring low-slope broadleaved and coniferous forests [55]. These findings highlight the similar habitat preferences between sables and yellow-throated martens. In terms of diet, Bao et al. discovered that sables primarily feed on mammals, birds, insects, and berries and seeds [22]. Zhu et al. observed that the diet of the yellow-throated marten consists of rodents and hares, birds, insects, and plants [56]. The diets of sables and yellow-throated martens exhibit some similarity, with the latter displaying slightly greater diversity. The shared habitat utilization and dietary preferences may result in spatial overlap between sables and yellow-throated martens.

If the intensity of interspecific competition becomes excessive, it will accelerate the depletion of natural resources, thus disrupting ecosystem balance [57]. The Schoener’s D index between the sable and yellow-throated marten was 0.56, indicating a relatively high degree of niche overlap in the two-dimensional environmental space quantified and constructed via principal component analysis following dimensionality reduction in DR, DS, Slope, and DCBMF. This means that there is a significant competitive interaction between them. The ecological behaviors of the sable and yellow-throated marten are similar, and the yellow-throated marten exhibits a larger body size (the sable weighs about 1 kg; the yellow-throated marten weighs approximately 2–3 kg), greater adaptability to the environment, and a more diverse diet. The yellow-throated marten lives in groups, and the sable lives alone. Theoretically, the yellow-throated marten should have an advantage in terms of competition, enabling it to occupy broader niches. However, we found the opposite; i.e., the sable occupied a larger range of niches than the yellow-throated marten (Figure 4A). We conjecture that yellow-throated martens monopolize the areas with more food availability and that sables have to travel further to obtain food in areas with less food availability. Therefore, the sable requires larger home ranges and niches but has lower food availability than the yellow-throated marten. We also posit that this may be related to the different survival strategies of the two species. The sable relies on a larger population size to exploit and sustain more resources to facilitate population development and persistence. In contrast, the yellow-throated marten secures adequate survival resources through its strong survival ability. This perspective aligns with the r-k selection theory in ecological evolution [58]; however, it is far from a typical r-k strategy. In future studies, we recommended exploring research opportunities in this field to provide new research directions on how to further differentiate species’ niches and offer novel avenues for differentiating species’ niches more effectively. 

The impact of human activities on wildlife is widespread throughout the world [59]. We observed that the first principal component (i.e., Dim1), which accounted for the largest total contribution (35.4%), was mainly related to DS and DR, both of which are closely intertwined with human activities. Studies have demonstrated that human activities exert a substantial influence on the quality of the habitats of the sable and yellow-throated marten [14]. To mitigate the impact of human interference on their survival and ensure that their habitats fulfill their essential needs, the sable and yellow-throated marten both chose areas far away from settlements. Concerning DR, it has been shown that roads have a barrier effect on wildlife, with large and medium-sized mammals significantly staying away from roads, and small mammals staying away from roads to a lesser extent compared to large and medium-sized mammals [60,61], which is consistent with our findings. There was substantial overlap between the sable and yellow-throated marten, but both did not keep away from roads. We think that the impact of roads on wildlife survival is primarily attributed to the intensity of disturbance from human activities, such as expressways, railways, and other high-traffic road types, which have a huge impact on animals because such roads lead to a reduction in wildlife habitat, as well as enhanced fragmentation, preventing communication between populations. Conversely, disused or lightly trafficked routes have a comparatively lesser impact on wildlife. Most of the roads in our study area were temporary roads constructed during logging. With large-scale logging being prohibited in the reserve in recent years, these roads have generally fallen into disuse, introducing minimal human disturbance. These roads introduce minimal resistance in terms of searching for prey [62,63,64]. It can be seen that the intensity of human activities plays a pivotal role in shaping the competitive niche of sables and yellow-throated martens. The research on the relationship between human activities and wildlife should be strengthened in future studies.

## 5. Conclusions

The suitable habitat for sables was larger than that for yellow-throated martens in the Taipinggou Nature Reserve, and there was a partial overlap of suitable habitats. The niches occupied by sables were larger than those occupied by yellow-throated martens, with a high degree of niche overlap between sables and yellow-throated martens. We suggest that the viability and resistance risk of the sable are weaker than those of the yellow-throated marten and that the survival strategies of species are important factors that influence coexistence between these species. In future studies, we can explore research on species coexistence from the perspective of survival strategies and the intensity of human activities. Our findings will contribute to the formulation of conservation and management policies for the sable and the yellow-throated marten. Based on our findings, we propose the following management and protection suggestions: (1) prioritize the conservation of suitable habitats while implementing targeted transformations for the remaining unsuitable habitats; (2) regulate human activities, such as vehicle usage, deforestation, and medicinal plant harvesting; and (3) according to survival characteristics, emphasize the further differentiation of ecological niches for the sable and the yellow-throated marten, to mitigate interspecific competition.

## Figures and Tables

**Table 1:** The information on 31 environmental variables. Accessed on 15 September 2023 for all webs below.

ID	Abbreviation	Name	Data Source
1	Slope	Slope	https://www.gscloud.cn/
2	DR	Distance from Road	https://www.webmap.cn/
3	DCBMF	Distance from Coniferous and Broadleaved Mixed Forest	https://www.resdc.cn/
4	DS	Distance from Settlements	https://www.webmap.cn/
5	Elevation	Elevation	https://www.gscloud.cn/
6	Aspect	Slope Aspect	https://www.gscloud.cn/
7	DT	Distance from Tributary	https://www.webmap.cn/
8	DMS	Distance from Main Stream	https://www.webmap.cn/
9	DM	Distance from Meadow	https://www.resdc.cn/
10	DBF	Distance from Broadleaved Forest	https://www.resdc.cn/
11	RDLS	Relief Degree of Land Surface	https://www.gscloud.cn/
12	NDVI	Normalized Difference Vegetation Index	https://www.resdc.cn/
13	Bio1	Annual Mean Temperature	https://www.worldclim.org/
14	Bio2	Mean Diurnal Range	https://www.worldclim.org/
15	Bio3	Isothermality	https://www.worldclim.org/
16	Bio4	Temperature Seasonality	https://www.worldclim.org/
17	Bio5	Max Temperature of Warmest Month	https://www.worldclim.org/
18	Bio6	Min Temperature of Coldest Month	https://www.worldclim.org/
19	Bio7	Temperature Annual Range	https://www.worldclim.org/
20	Bio8	Mean Temperature of Wettest Quarter	https://www.worldclim.org/
21	Bio9	Mean Temperature of Driest Quarter	https://www.worldclim.org/
22	Bio10	Mean Temperature of Warmest Quarter	https://www.worldclim.org/
23	Bio11	Mean Temperature of Coldest Quarter	https://www.worldclim.org/
24	Bio12	Annual Precipitation	https://www.worldclim.org/
25	Bio13	Precipitation of Wettest Month	https://www.worldclim.org/
26	Bio14	Precipitation of Driest Month	https://www.worldclim.org/
27	Bio15	Precipitation Seasonality	https://www.worldclim.org/
28	Bio16	Precipitation of Wettest Quarter	https://www.worldclim.org/
29	Bio17	Precipitation of Driest Quarter	https://www.worldclim.org/
30	Bio18	Precipitation of Warmest Quarter	https://www.worldclim.org/
31	Bio19	Precipitation of Coldest Quarter	https://www.worldclim.org/

**Table 2:** SRSS of environment variables and corresponding screening results.

ID	Name	SRSS	First and Second Principal Component Cumulative Variance Contribution	Third Principal Component Eigenvalue	Variable Selection
1	Slope	33.4	—	—	Yes
2	DR	23.8	—	—	Yes
3	DCBMF	16.1	—	—	Yes
4	DS	15.1	60.87%	0.98	Yes
5	Bio15	12.3	56.86%	1.01	No
6	Bio13	10.9	57.47%	1.01	No
7	Bio3	10.9	54.99%	1.14	No
8	Elevation	6.4	56.02%	1.19	No
9	Aspect	4.5	49.83%	1.23	No
10	Bio2	4.4	53.04%	1.25	No
11	DT	3.8	48.26%	1.55	No
12	Bio1	3.5	50.43%	1.56	No
13	DMS	3.1	50.26%	1.59	No
14	DM	3.0	48.42%	1.76	No
15	Bio12	2.1	49.14%	1.83	No
16	NDVI	2.0	48.49%	1.84	No
17	Bio9	1.6	47.81%	1.94	No
18	Bio14	1.3	46.49%	1.99	No
19	DBF	0.3	44.42%	2.05	No

## Data Availability

The environment variables for this study can be found in Geospatial Data Cloud (https://www.gscloud.cn/), National Catalogue Service For Geographic Information (https://www.webmap.cn/), Resource and Environmental Science Data Platform (https://www.resdc.cn/), and WorldClim (https://www.worldclim.org/). Accessed on 15 September 2023 for all webs above. The R code can be found in the Supplementary Materials. The occurrence sites for the sable and yellow-throated marten can be obtained by contacting the author (hy1624@126.com).

## Supplementary Materials

The following supporting information can be downloaded at: https://www.mdpi.com/article/10.3390/biology13080594/s1, Table S1: Contribution and importance of environmental variables for modeling the sable and yellow-throated marten, and the R code used in this study.
