# Supplementary material for: Suitable Habitat Distribution and Niche Overlap of the Sable (Martes zibellina) and Yellow-Throated Marten (Martes flavigula) in Taipinggou National Nature Reserve, Heilongjiang Province, China
Source: Biology (Basel). 2024 Aug 7;13(8):594. doi: 10.3390/biology13080594 (PMC11351473; doi:10.3390/biology13080594)
Supplement: Supplementary file 1 [file biology-13-00594-s001.zip › biology-3124180-supplementary.pdf]

Table S1. Contribution and importance of environmental variables for modelling the sable and yellow-throated marten.

**Sigillum**	Name	Sable		Yellow Throat Marten	
		contribution	importance	contribution	importance
**Elevation**	Elevation	2.5	6.3	0.9	0.8
Slope	Slope	27.3	26.9	10.8	19.8
Aspect	Slope aspect	0.7	0.7	7.8	4.4
DR	Distance from Road	17.3	21.3	18	10.7
DS	Distance from Settlements	8.1	5.1	9	14.2
DMS	Distance from the Main Stream	15.8	3	0.3	0.7
DT	Distance from Tributaries	1.9	3.2	2	2
DM	Distance from Meadows	2.5	2.7	3.1	1.4
DBF	Distance from Broad-leaved Forest	0.6	0.1	0	0.3
DCBMF	Distance from Coniferous and Broadleaved Mixed Forest	5	5.7	13.5	15.1
NDVI	Normalized Difference Vegetation Index	2.5	2	0.2	0.2
Bio1	Annual Mean Temperature	1.1	0.6	2.2	3.4
Bio2	Mean Diurnal Range	1.6	4.2	12.7	1.4
Bio3	Isothermality	2.8	10.8	4.2	1.1
Bio9	Mean Temperature of Driest Quarter	2.3	1.6	0	0.1
Bio12	Annual Precipitation	1.2	2.1	0.1	0.3
Bio13	Precipitation of Wettest Month	1.5	1.1	5.6	10.8
Bio14	Precipitation of Driest Month	3	0.6	1.7	1.2
Bio15	Precipitation Seasonality	2.4	2	7.9	12.1

The R code used in this study

```
install.packages("sp")
install.packages("raster")
install.packages("dismo",type = "binary")
library(sp)
library(raster)
library(dismo)
path1 <-
"C:/Users/123456/Desktop/Data_processing/MaxEnt/huanghoudiao30_20%/1/test/qxd_feishiyi.tif"
file1 <- raster(path1)
set.seed(1963)
background <- randomPoints(file1, 1000)
result <-
write.csv(background,"C:/Users/123456/Desktop/Data_processing/PCA/huanghoudiao/huanghoudiao_absence.csv",row.names = FALSE)


library(terra)
library(ecospat)
library(ade4)
```

```
library(base)
file_path1                                                                              <-
"C:/Users/123456/Desktop/Data_processing/PCA/zidiao/zidiao_background_TableToExcel_E
ng.csv"
zidiao_data <-read.csv(file_path1,header = TRUE,sep = ",")
zidiao_data
file_path2                                                                              <-
"C:/Users/123456/Desktop/Data_processing/PCA/huanghoudiao/huanghoudiao_background
_TableToExcel_Eng.csv"
huanghoudiao_data <-read.csv(file_path2,header = TRUE,sep = ",")
huanghoudiao_data


pca.env <- dudi.pca(rbind(zidiao_data,huanghoudiao_data)[,5:8],scannf=F,nf=2)

TSS <- function(
    cont1,
    cont2 = NULL,
    presAbs,
    thresh1,
    thresh2,
    largerPres1 = TRUE,
    largerPres2 = FALSE,
    link = `&`
    ) {
    presAbs <- as.logical(presAbs)
    if (is.null(cont2)) {
        sel <- (!is.na(cont1)) & (!is.na(presAbs))
        cont1 <- cont1[sel]
        presAbs <- presAbs[sel]
        thresh2 <- NA
    } else {
        sel <- (!is.na(cont1)) & (!is.na(cont2)) & (!is.na(presAbs))
        cont1 <- cont1[sel]
        cont2 <- cont2[sel]
        presAbs <- presAbs[sel]
    }
    thresh <- expand.grid(thresh1, thresh2)
    ##
    n <- sum(presAbs) + sum(!presAbs)
    n2 <- n^2

    no <- rep(NA, nrow(thresh))
    result <- list(
```

```
        threshold1 = no,
        threshold2 = no,
        overallAccuracy = no,
        sensitivity = no,
        specificity = no,
        tss = no,
        kappa = no,
        pP = no,
        pA = no,
        aA = no,
        aP = no,
        n = n
        )


    for ( i in 1:nrow(thresh)) {
        ## Classify cont1 and cont2 into absence (< tr)
        ## and presence (>= tr) depending on largerPres1
        ## and largerPres2
        result$threshold1[i] <- thresh[i, 1]
        if (largerPres1) {
            vecCont1 <- cont1 >= thresh[i, 1]
        } else {
            vecCont1 <- cont1 <= thresh[i, 1]
        }
        if (!is.null(cont2)) {
            if (largerPres2) {
                vecCont2 <- cont2 >= thresh[i, 2]
            } else {
                vecCont2 <- cont2 <= thresh[i, 2]
            }
            ## Combine vecCont1 and vecCont2 using "link"
            vecCont <- link(vecCont1, vecCont2)
            result$threshold2[i] <- thresh[i, 2]
        } else {
            vecCont <- vecCont1
        }

        ## Presence predicted and Present
        pP <- sum(      vecCont    &      presAbs    ) # a
        result$pP[i] <- pP
        ## Presence predicted but absent
        pA <- sum(      vecCont    & (!presAbs) ) # b
        result$pA[i] <- pA
```

```
        ## Absence predicted but Present
        aP <- sum( (!vecCont) &      presAbs    ) # c
        result$aP[i] <- aP
        ## Absence predicted and Absent
        aA <- sum( (!vecCont) & (!presAbs) ) # d
        result$aA[i] <- aA

        ## -------------------------
        ## Sensitivity
        Sens <- pP / (pP + aP)
        result$sensitivity[i] <- Sens
        ## -------------------------
        ## Specificity
        Spe <- aA / (aA + pA)
        result$specificity[i] <- Spe
        ## -------------------------
        ## TSS
        TSS <- Sens + Spe - 1
        result$tss[i] <- TSS
        ## -------------------------
        ## Overall Accuracy
        ovAc <- (aA + pP) / n
        result$overallAccuracy[i] <- ovAc
        ## -------------------------
        ## kappa
        ## Should this be implemented?
        t1 <- (pP + aA) / n
        t2 <- ( (pP + pA)*(pP + aP)+(aP + aA)*( aA + pA) ) / n2
        kap <- (t1 - t2) / (1 - t2)
        result$kappa[i] <- kap
        ## -------------------------
    }
    ##
    class(result) <- "TSS"
    attr(result, "link") <- link
    attr(result, "largerPres1") <- largerPres1
    attr(result, "largerPres2") <- largerPres2
    attr(result, "threshold1") <- thresh1
    attr(result, "threshold2") <- thresh2
    attr(result, "dimension") <- ifelse(is.null(cont2), 1, 2)
    return(result)
}
```

```r
library("FactoMineR")
library("factoextra")
library("ggplot2")
eig.val <- get_eigenvalue(pca.env)
eig.val
var <- get_pca_var(pca.env)
var
head(var$coord, 7)
head(var$cos2, 7)
fviz_pca_var(pca.env, col.var = "cos2",
             gradient.cols = c("#00AFBB", "#E7B800", "#FC4E07"),
             repel = TRUE)
scores.globclim <- pca.env$li
scores.zidiao.nat <- suprow(pca.env,zidiao_data[which(zidiao_data[,24]==1),5:8])$li
scores.huanghoudiao.nat <-
suprow(pca.env,huanghoudiao_data[which(huanghoudiao_data[,24]==1),5:8])$li
scores.clim.zidiao <- suprow(pca.env,zidiao_data[,5:8])$li
scores.clim.huanghoudiao <- suprow(pca.env,huanghoudiao_data[,5:8])$li


grid.clim.zidiao <- ecospat.grid.clim.dyn(glob=scores.globclim,
                                          glob1=scores.clim.zidiao,
                                          sp=scores.zidiao.nat,
                                          R=100,th.sp=0)
grid.clim.huanghoudiao <- ecospat.grid.clim.dyn(glob=scores.globclim,
                                          glob1=scores.clim.huanghoudiao,
                                          sp=scores.huanghoudiao.nat,
                                          R=100,th.sp=0)


D.overlap <- ecospat.niche.overlap (grid.clim.zidiao, grid.clim.huanghoudiao, cor=T)$D
D.overlap


niche.dyn <- ecospat.niche.dyn.index (grid.clim.zidiao, grid.clim.huanghoudiao, intersection =
0.1)
ecospat.plot.niche.dyn(grid.clim.zidiao, grid.clim.huanghoudiao, quant=0.5, interest=2,
                       title= "Niche Overlap", name.axis1="Dim1",
                       name.axis2="Dim2")

grid.clim.podu.zidiao <-
ecospat.grid.clim.dyn(glob=as.data.frame(rbind(zidiao_data,huanghoudiao_data)[,5]),
                                          glob1=as.data.frame(zidiao_data[,5]),
```

```
sp=as.data.frame(zidiao_data[which(zidiao_data[,24]==1),5]),
                                    R=1000, th.sp=0)
grid.clim.podu.huanghoudiao <-
ecospat.grid.clim.dyn(glob=as.data.frame(rbind(zidiao_data,huanghoudiao_data)[,5]),

glob1=as.data.frame(huanghoudiao_data[,5]),

sp=as.data.frame(huanghoudiao_data[which(huanghoudiao_data[,24]==1),5]),
                                         R=1000, th.sp=0)
podu.dyn<-ecospat.niche.dyn.index (grid.clim.podu.zidiao, grid.clim.podu.huanghoudiao,
#                              intersection=0.1)
ecospat.plot.niche.dyn(grid.clim.podu.zidiao, grid.clim.podu.huanghoudiao,
quant=0.5,interest=2, title= "Niche Overlap",
                    name.axis1="Slope")


grid.clim.podu.zidiao <-
ecospat.grid.clim.dyn(glob=as.data.frame(rbind(zidiao_data,huanghoudiao_data)[,6]),
                                         glob1=as.data.frame(zidiao_data[,6]),

sp=as.data.frame(zidiao_data[which(zidiao_data[,24]==1),6]),
                                         R=1000, th.sp=0)
grid.clim.podu.huanghoudiao <-
ecospat.grid.clim.dyn(glob=as.data.frame(rbind(zidiao_data,huanghoudiao_data)[,6]),

glob1=as.data.frame(huanghoudiao_data[,6]),

sp=as.data.frame(huanghoudiao_data[which(huanghoudiao_data[,24]==1),6]),
                                              R=1000, th.sp=0)
ecospat.plot.niche.dyn(grid.clim.podu.zidiao, grid.clim.podu.huanghoudiao,
quant=0.5,interest=2, title= "Niche Overlap",
                    name.axis1="DR")

grid.clim.podu.zidiao <-
ecospat.grid.clim.dyn(glob=as.data.frame(rbind(zidiao_data,huanghoudiao_data)[,7]),
                                         glob1=as.data.frame(zidiao_data[,7]),

sp=as.data.frame(zidiao_data[which(zidiao_data[,24]==1),7]),
                                         R=1000, th.sp=0)
grid.clim.podu.huanghoudiao <-
ecospat.grid.clim.dyn(glob=as.data.frame(rbind(zidiao_data,huanghoudiao_data)[,7]),

glob1=as.data.frame(huanghoudiao_data[,7]),
```

```
sp=as.data.frame(huanghoudiao_data[which(huanghoudiao_data[,24]==1),7]),
                                        R=1000, th.sp=0)
ecospat.plot.niche.dyn(grid.clim.podu.zidiao,            grid.clim.podu.huanghoudiao,
quant=0.5,interest=2, title= "Niche Overlap",
                name.axis1="DCBMF")

grid.clim.podu.zidiao                                                          <-
ecospat.grid.clim.dyn(glob=as.data.frame(rbind(zidiao_data,huanghoudiao_data)[,8]),
                                    glob1=as.data.frame(zidiao_data[,8]),

sp=as.data.frame(zidiao_data[which(zidiao_data[,24]==1),8]),
                                    R=1000, th.sp=0)
grid.clim.podu.huanghoudiao                                                    <-
ecospat.grid.clim.dyn(glob=as.data.frame(rbind(zidiao_data,huanghoudiao_data)[,8]),

glob1=as.data.frame(huanghoudiao_data[,8]),

sp=as.data.frame(huanghoudiao_data[which(huanghoudiao_data[,24]==1),8]),
                                        R=1000, th.sp=0)
ecospat.plot.niche.dyn(grid.clim.podu.zidiao,            grid.clim.podu.huanghoudiao,
quant=0.5,interest=2, title= "Niche Overlap",
                name.axis1="DS")
```
